# Current Management of Diaphyseal Long Bone Defects—A Multidisciplinary and International Perspective

**DOI:** 10.3390/jcm12196283

**Published:** 2023-09-29

**Authors:** Steffen Bernd Rosslenbroich, Chang-Wug Oh, Thomas Kern, John Mukhopadhaya, Michael Johannes Raschke, Ulrich Kneser, Christian Krettek

**Affiliations:** 1 Department of Trauma, Hand and Reconstructive Surgery, University Hospital Muenster, 48149 Münster, Germany; michael.raschke@ukmuenster.de; 2 Department of Orthopedic Surgery, School of Medicine, Kyungpook National University, Kyungpook National University Hospital, Jung-gu, Daegu 41944, Republic of Korea; cwoh@knu.ac.kr; 3 Department of Trauma Surgery/Murnau, BG Unfallklinik Murnau, 82418 Murnau am Staffelsee, Germany; thomas.kern@bgu-murnau.de; 4 Orthopedic and Trauma Department, Paras HMRI Hospital, Patna 800014, Bihar, India; mukhoj@gmail.com; 5 BG Trauma Center Ludwigshafen, Department of Plastic Surgery, University of Heidelberg/Ludwigshafen, 67059 Heidelberg, Germany; ulrich.kneser@bgu-ludwigshafen.de; 6 Trauma Department/Hannover, Hannover Medical School, 30625 Hannover, Germany; krettek.christian@mh-hannover.de

**Keywords:** bone defect, callus distraction, all-internal distraction, Ilizarov

## Abstract

The treatment of defects of the long bones remains one of the biggest challenges in trauma and orthopedic surgery. The treatment path is usually very wearing for the patient, the patient’s environment and the treating physician. The clinical or regional circumstances, the defect etiology and the patient´s condition and mental status define the treatment path chosen by the treating surgeon. Depending on the patient´s demands, the bony reconstruction has to be taken into consideration at a defect size of 2–3 cm, especially in the lower limbs. Below this defect size, acute shortening or bone grafting is usually preferred. A thorough assessment of the patient´s condition including comorbidities in a multidisciplinary manner and her or his personal demands must be taken into consideration. Several techniques are available to restore continuity of the long bone. In general, these techniques can be divided into repair techniques and reconstructive techniques. The aim of the repair techniques is anatomical restoration of the bone with differentiation of the cortex and marrow. Currently, classic, hybrid or all-internal distraction devices are technical options. However, they are all based on distraction osteogenesis. Reconstructive techniques restore long-bone continuity by replacing the defect zone with autologous bone, e.g., with a vascularized bone graft or with the technique described by Masquelet. Allografts for defect reconstruction in long bones might also be described as possible options. Due to limited access to allografts in many countries and the authors’ opinion that allografts result in poorer outcomes, this review focuses on autologous techniques and gives an internationally aligned overview of the current concepts in repair or reconstruction techniques of segmental long-bone defects.

## 1. Introduction

Defects of the long bones are challenging for the patient, the patient´s environment and the treating physician. Depending on the clinical or regional circumstances, the distribution of the defect etiology differs. The main reasons for defects of the long bones are the following:

- traumatic substance loss due to open fracture or debridement
- fracture-associated infection or osteomyelitis
- nonunion
- tumor

Defects with a defect size of up to 2–3 cm are usually treated with grafting or acute shortening. Defects larger than 3 cm, especially in the lower limbs, are frequently restored to normal to avoid further functional impairment. A thorough assessment of the patient´s condition including comorbidities in a multidisciplinary manner and her or his personal demands must be taken into consideration. The mental stability and the strength of the patient to collaborate in these often very exhausting treatments are frequently underestimated. These considerations, the experience of the treating surgeon and the healthcare circumstances define the treatment path. Several techniques are available to restore continuity of the long bone. These techniques can be used alternatively or subsequently. Knowledge of all options and their individual pros and cons might be helpful for the physician to avoid problems and obstacles in these lengthy limb-saving procedures. In general, these techniques can be divided into *repair* techniques and *reconstructive* techniques.

The aim of the repair techniques is anatomical restoration of the bone with differentiation of the cortex and marrow. The basis for these techniques is distraction osteogenesis, which was influenced by names such as Langenbeck, Codovilla, Bier and Magnussen. The milestone work by Ilizarov in terms of instrument development and scientific research made distraction osteogenesis available for modern medicine. The term mechanotransduction was defined by his work that described the cellular mechanism for bone adaptation to mechanical loading, resembling the foundation for distraction osteogenesis.

Currently, classic, hybrid or all-internal distraction devices are technical options. However, they are all based on gradual distraction and consecutive bone transport or limb lengthening in osseous defect situations.

Reconstructive techniques restore long-bone continuity by replacing the defect zone with autologous bone, e.g., with a vascularized bone graft or with the technique described by Masquelet. Allografts for defect reconstruction in long bones might also be described as possible options. Due to the limited access to allografts in many countries and the authors’ opinion that allografts result in poorer outcomes, this review focuses on autologous techniques and gives an internationally aligned overview of the current concepts in repair or reconstruction techniques of segmental long-bone defects.

### 1.1. Classic Bone Transport

**Figure 1:**
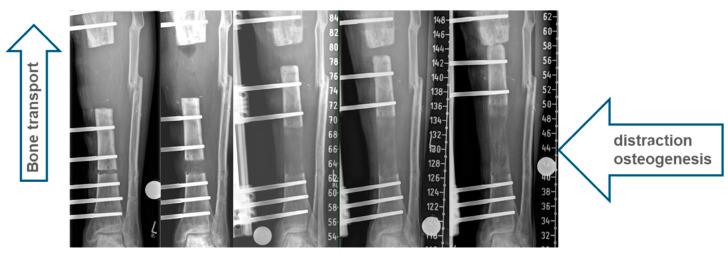
Distraction osteogenesis as classic segmental bone transport in the left tibia.

Bone transport is defined as the gradual relocation of a bone segment from a healthy area to a region of bone loss and regeneration by distraction osteogenesis (Figure 1). Many small steps were necessary before the classical bone-transport method was developed. As early as the mid-19th century, Bernhard von Langenbeck described that the longitudinal growth of bones could be increased by distraction [1].

Léopold Ollier recognized the importance of the periosteum for bone growth [2]. Even though Alesandro Codivilla had already gained experience with limb lengthening in 1905, those attempts were usually associated with very high complication rates [3]. Louis Ombrédanne was the first to use slow–gradual lengthening for the first time [4], and Vittorio Putti improved the fixator technique [5]. August Bier recognized the importance of hematoma for bone healing [6]. The latency period after osteotomy to start lengthening was pioneered by Leroy Abbott [7]. Drilling osteoclasis was first described by Max Brandes [8], and Bosworth named the procedure bone distraction [9].

Raimund Wittmoser devised a ring fixator in 1944, but Lorenz Boehler did not recognize the brilliance and forbade him from publishing those ideas [10]. In 1951, Pierre Bertrand first used intramedullary nails for stabilization for femoral distraction [11]. Heinz Wagner recognized the importance of early mobilization and, therefore, developed a more stable fixator system [12]. After isolation by the Iron Curtain, Gavriil Ilizarov developed the method of bone transport. In 1952, he received a Russian patent for his ring fixator and then published “Compression osteosynthesis with the author’s device” in 1968. He performed many experimental studies on the biology of bone formation and developed a method for bone transport by callus distraction [13,14,15,16].

The Ilizarov ring fixator has been available in the West only since 1981. 

The foundation for all repair techniques is the technique of distraction osteogenesis, a low-energy osteotomy is performed with gentle drilling (osteoclasia without heat) or a gigli saw, preferably at the proximal or distal part of the affected bone at the metaphyseal area. The focus should be on minimal soft-tissue and periosteal compromise to preserve blood supply. At the osteotomy site, the hematoma changes to a viscous callus that can be distracted after a latency period of 3–7 days. Ilizarov described the optimal speed of distraction as 1 mm/day in four steps. Depending on the patient’s condition and the soft-tissue situation and radiographic callus formation, the speed of distraction is individually modified. Raschke described intramedullary bone transport combined with a monolateral fixation system 30 years ago [17]. Since then there was a strong development of new instruments external and internal which are discussed throughout this review.

Segmental transport can be performed with various devices, such as ring/circular fixators, monolateral fixators and intramedullary nail systems, or by combinations of more than one device. Each device or technique has its own advantages and disadvantages. For bone transport, a stabilization system and a motor are needed.

We define “classic bone transport” as procedures in which only external devices are used. On the femur, problems arise because of the large soft tissue cover; monolateral systems become unstable, and ring fixators become uncomfortable. That is why we only use classic bone transport on the femur in special cases.

**Figure 2:**
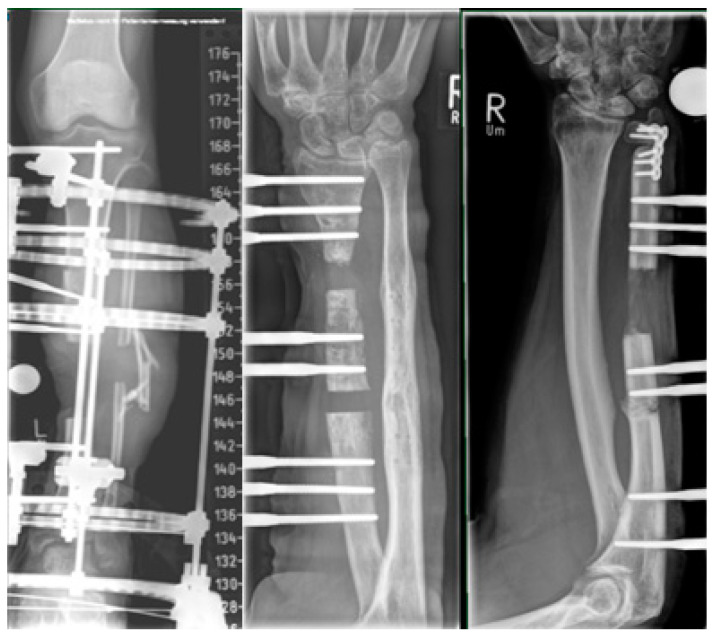
Classic segmental bone transport in the left tibia, right radius and right ulna.

We observe an indication for classic bone transport, especially at the tibia, radius and ulna (Figure 2).

To use a monolateral fixator, sufficiently long bone fragments are needed. This usually works for diaphyseal defects. If we have short bone ends, a classic Ilizarov ring fixator must be used.

If the joint line at one end of the bone is completely missing, and docking arthrodesis is required, the ring fixator is a possible way to solve the problem.

**Figure 3:**
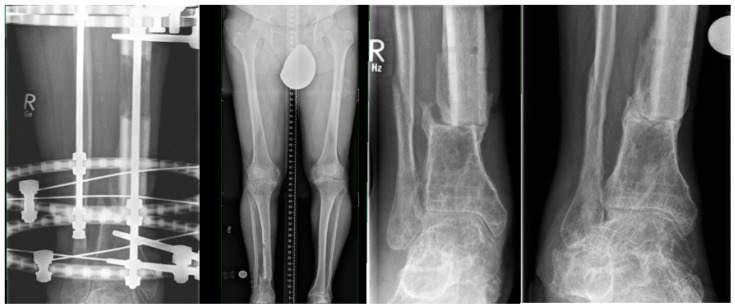
Translation with remodeling after docking in the course of consolidation.

In the case of plastic reconstruction, cable systems can be used when a flap forbids us from pulling wires through it. Cable systems are also suitable for long transport distances, as the transport fragment then arrives at the docking point without translation (Figure 3).

Although patients rarely ask for an external fixator, we still observe classic bone transport as an indispensable method for many cases. Despite the risks associated with the long time that an external fixator has to be in place, it is a safe procedure with reproducibly good results.

**Figure 4:**
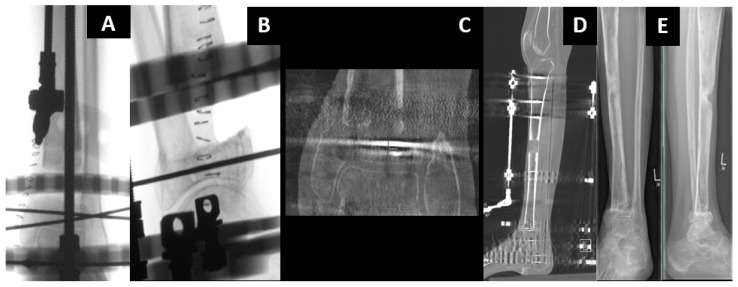
Example of classic bone transport.

Figure 4A–E show a case that could hardly be solved in a joint-preserving manner without classical bone transport in a ring fixator.

After a pilon tibial fracture, the joint section had healed, but osteomyelitis developed directly above it. After definitive treatment of the infection, only an 8 mm thin slice of distal tibia remained. We built up a ring fixator across the ankle joint and fixed the pilon fragment with two wires (Figure 4A,B). A gentle drilling osteoclasia (without heat) produced a transport segment in the proximal part. Six to nine days after surgery, transport began three times 0.25 mm per day. In the subsequent process, the distraction speed can be adjusted depending on the radiographic callus formation. When the transport segment arrives (Figure 4C,D), surgical docking with autologous bone grafting and compression can reduce the time to fixator removal. When the docking zone has healed and the regenerate has hardened, the fixator can be removed (Figure 4E). During the entire process, the leg axis, length and torsion must be controlled.

### 1.2. Hybrid Techniques of Segmental Bone Transport

**Figure 5:**
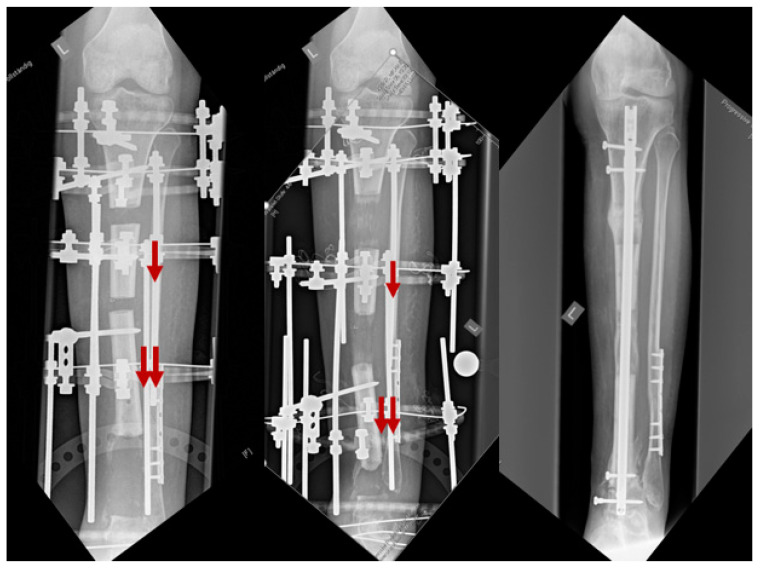
A thirty-year-old female patient after post-traumatic osteomyelitis and resection of the distal tibia received multifocal bone transport (tandem-technique), doubling distraction speed of the distal fragment. After docking, the approach was changed to an antibiotic-coated nail for consolidation and partial weight bearing. Red arrows show the distraction direction—the segment with the double arrows is transported with twice the speed.

Classic bone transport using an external fixator (EF) has many advantages, including an unlimited amount of bone regeneration, the capacity to correct the deformity and early weight bearing. However, it requires a long period of external fixation, including the distraction period, healing at the docking site and consolidation of the distraction callus. Therefore, avoiding complications, such as pin-related problems and joint contractures, is difficult and can result in poor outcomes [18]. Although multifocal bone transport techniques (Figure 5) have reduced the distraction period, consolidation of the distraction callus may still require a long period until it is safe to remove the EF. Therefore, secondary nailing that follows the distraction phase might be an option.

Unless the distraction callus is sufficiently hard, EF removal may result in a fractured callus, nonunion of the docking site, malunion or even ultimate failure of bone transport. To reduce the external fixation time (EFT) as well as its resultant complications, hybrid bone-transport techniques have been developed. A combination of an EF and an internal implant (either a nail or a plate) can be performed simultaneously, while the EF can be removed early before completion of the consolidation. With its mechanical advantage to support the distraction callus and protect it against refracture, it may help the patient´s comfort and convenience.

Bone transport over a nail (BTON) is a commonly used hybrid technique that eliminates the consolidation period of the distraction callus with the stabilization provided by the intramedullary (IM) nail. BTON remarkably reduced EFT, which was associated with increased patient comfort, a decreased complication rate and convenient and rapid rehabilitation [19]. However, the conventional BTON technique should maintain EF until docking-site consolidation is completed. Therefore, modifications of BTON have been attempted to achieve stability and compression at the docking site by performing additional plate fixation or by locking the transported segment to the predrilled, custom-made, intramedullary nail via the extra locking holes [20]. BTON has a potential risk of developing a deep infection in the medullary canal. As close proximity is inevitable between the nail and EF pins, cross-contamination from the infected pin track may lead to bone reconstruction failure [21].

Bone transport over a plate (BTOP) combines plate fixation with EF. Compared with BTON, BTOP may decrease the risk of deep infection because it can minimize possible contact between the plate and EF pins [22]. Since the transported fragment is fixed to the plate with screws at the time of docking, BTOP requires the external fixator only during the distraction period, eliminating both the consolidation periods of the docking site and the distraction callus. A recent comparative study proved that BTOP had a significantly shorter EFT than BTON, while the final outcomes were similar in segmental tibial bone defects [23]. Nail fixation is also difficult when the defect is too close to the joint line and when the proximal or distal segment is too short for nail locking. BTON may be difficult to perform in forearm bones because of a very limited intramedullary space between the nail and EF. Under these conditions, BTOP is an ideal alternative since plate fixation is relatively free without anatomical restrictions. In summary, both hybrid bone-transport techniques are safe and achieve satisfactory outcomes for treating segmental defects of the long bone. The BTOP technique shows benefits over the BTON technique because of the shorter EFT and wider indications; however, in the lower leg, more stability is achieved with BTON.

**Figure 6:**
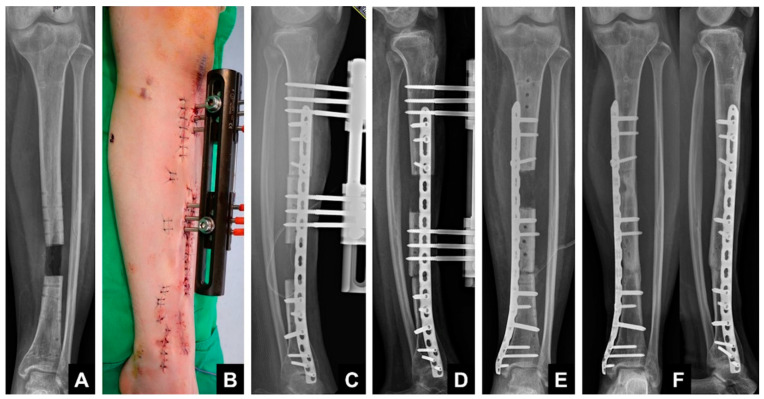
Example of hybrid bone transport.

A 15-year-old girl suffered reaming necrosis of the tibial shaft after tibial nailing (Figure 6A). A complete resection of necrotic bone resulted in a 3.5 cm segmental defect (Figure 6B). The BTOP procedure was performed. Figure 6C shows the lateral radiograph immediately after locked plate insertion on the medial aspect, corticotomy and application of an EF on the anterior aspect. Figure 6D shows the lateral radiograph after the completion of bone transport. Figure 6E shows the AP radiograph after screw fixation at the transported segment, autogenous iliac bone grafting at the docking site and removal of EF. Figure 6F shows the AP and lateral radiograph of the tibia showing bony healing of the docking site and distraction site 1 year after transport.

### 1.3. Induced Membrane Technique—Masquelet

Membrane-induced osteogenesis, first reported by Masquelet in 2000, is a popular technique for the reconstruction of bone defects [24]. Among thirty-five patients with defects from 4 cm to 25 cm, union rates of 100% were reported. It has revolutionized our understanding of bone healing and has become a popular method of treating bone defects. Subsequently, many authors have reported on the success of this technique [25,26,27,28,29].

### 1.4. Principle

The bone defect is filled with cement (polymethylmethacrylate), which provokes a foreign-body reaction that results in the formation of a strongly vascularized membrane. This later functions as a biological chamber that can be filled with autologous bone grafts, resulting in solid cylindrical bone formation. The membrane prevents graft absorption and promotes bone healing via several vascular and growth factors.

### 1.5. Technique

The technique is a two-stage procedure. In the first stage, all nonviable bone and soft tissues are debrided. This is possibly the most important stage in the procedure. The limb needs to be stabilized, and the defect is filled with bone cement mixed with antibiotics, overlapping the cortices at the two ends. Good soft tissue cover is essential.

In the second stage, the membrane is incised, and the cement is removed. The medullary canals at the two ends are opened, the defect is filled with cancellous bone graft, and the membrane closes over it along with the soft tissue.

Masquelet in his series used external fixation for stabilization and cancellous autografts. However, other implants, such as interlocking nails [29,30], may be used. Similarly, authors have combined autologous bone grafts with bone substitutes, such as demineralized bone matrix or BMP [31], or used reamer-irrigation-aspirator (RIA) bone grafts [30,32].

Many articles have been written on the details of the technique using minor modifications [33,34,35,36,37,38].

## 2. Membrane Characteristics

The biologic activity of the induced membrane has been studied extensively. Pellesier et al. [37] showed that the induced membrane secreted growth factors, including vascular and osteoinductive factors, which could stimulate bone regeneration. Authors have studied the histologic and biochemical properties of the membrane in animal studies and have shown that, within two weeks, the membrane forms two distinct layers. Most studies have shown a significant increase in various factors that promote osteogenesis [37,39,40,41].

The vascularity of the outer part is more than that of the part in contact with the cement spacer and progressively increases from 2 to 4 weeks and decreases after 4–6 weeks.

Aho et al. [39] have shown that the membrane from human femur or tibia defects is significantly less vascular at 3 months than at 1 month.

Spacer Material

Masquelet used PMMA to fill the defect after debridement. The addition of antibiotics should help to combat infection due to the elution of high concentrations of local antibiotics [42,43,44,45].

Nau et al. [40] showed that different antibiotics may affect the membrane characteristics and that clindamycin produced a thinner membrane than vancomycin or gentamycin.

PMMA is conveniently available and most commonly used in clinical practice [46]. Alternative spacers, such as silicone epoxies and biosynthetic materials, may have a role in the future [47].

Bone graft materials

Autogenous cancellous bone grafting is still the gold standard. RIA has become popular more recently because of the large volume of grafts with reduced complications. Bone substitutes, such as hydroxyapatite, may have benefits in increasing graft volume but not osteogenesis. BMP may help increase osteogenic properties, but clinical trials are still equivocal [31].

In conclusion, this method is very popular due to the excellent reports in complex situations. The renerate however, has a column-like structure, which might require further protection with an implant (e.g., plate or nail) until full weight bearing can be permitted.

**Figure 7:**
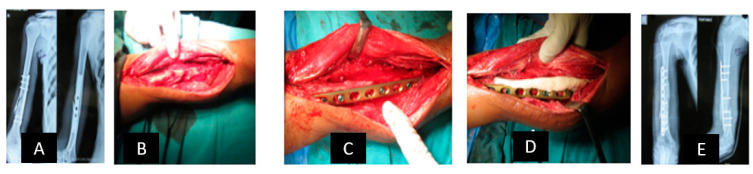
A 19-year-old patient was referred with swelling induration and sinus discharge 6 months after operative management of a humerus shaft fracture. (**A**) Preoperative X-ray: AP and lateral view. (**B**) Intraoperative photographs showing purulent discharge. (**C**) Through debridement excision of sequestrated bone stabilization with a locking compression plate (LCP) using locking screws only. (**D**) Filling of the bone defect with antibiotic-impregnated bone cement. (**E**) AP and lateral views after stage one.

**Figure 8:**
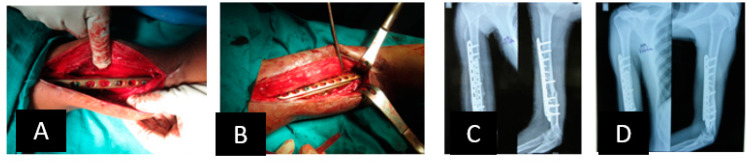
After six weeks, membrane formation was induced after removal of the cement spacer in stage two. (**A**,**B**) The defect was filled with a bone graft and an additional orthogonal plate for enhanced rotational stability. (**C**) Subsequent postoperative X-ray, AP and lateral views. (**D**) AP and lateral radiographs at the one-year follow-up showed successful healing.

Figure 7 and Figure 8 show an example of the induced membrane technique—Masquelet.

Stage One:

Stage Two:

### 2.1. Reconstruction with Vascularized Bone Graft

Vascularized bone grafts are an additional option for extensive bone defects or bone defects with impaired regenerative potential that require simultaneous structural support, bone regeneration and vascularization. In contrast to conventional bone grafts, vascularized bone grafts allow for direct healing and bridging of bony defects and do not require a process of partial necrosis or regeneration (creeping substitution) [48]. Over the last 40 years, a plethora of different vascularized bone grafts have been described for the reconstruction of bone defects in any region of the human body, including the extremities, trunk and head and neck [49]. Since its first description in 1975 by Taylor et al. [50], the free fibula graft has become the working horse flap for the reconstruction of large segmental bone defects. It is a long and straight graft and can be raised with a skin island that allows for the repair of concomitant soft-tissue defects. Donor-site morbidity is tolerable if 6–7 cm of the distal and proximal fibula is preserved [51]. Stress fractures are not uncommon after reconstruction of lower extremity bone defects with the free fibula graft, and it can sometimes take more than one year for graft hypertrophy to allow for full weight-bearing mobility [52]. At the lower extremities, the combination of free vascularized fibula grafts and allogenic bone grafts combines high mechanical stability and osteogenic potential and provides safe and stable healing in aseptic bone defects [53]. Shorter bone defects can also be addressed with a “double barrel” fibula graft for increased stability [54]. Free iliac crest [55] and scapula grafts [56] represent alternative options that are applicable in shorter defects or if the fibula graft is not available. While bone transport and membrane-guided bone regeneration are alternatives to free vascularized bone grafts at the lower extremities, free vascularized bone grafts represent the gold standard for segmental bone defects at the upper extremities due to their healing potential, mechanical properties and single-stage surgery that allow for early mobilization and physical therapy.

**Figure 9:**
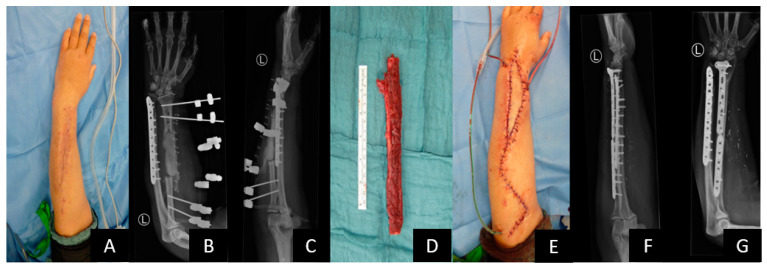
Example of vascularized bone graft. (**A**–**C**) Local wound situation and X-ray before reconstruction. Segmental bone defect is filled with a gentamycin spacer and stabilized with external fixation. (**D**) Free fibula graft prior to inset. (**E**) Primary closure with the skin island of the osteocutaneous fibula flap. (**F**,**G**) Stabile bony union at 12 months after reconstruction.

A 50-year-old female patient with a 3rd degree open radius and ulna fracture, showing ORIF radius and ulna at day 0; revision and cancellous bone grafting at days 24 and 63; severe bone and soft tissue infection, wound revision and sequestrectomy at day 75; and radius segment resection, spacer and external fixator at day 94 (Figure 9A–C). Reconstruction of an 8 cm segmental radius defect with free vascularized osteocutaneous fibula graft (Figure 9D). and ORIF (2 LCP plates) was performed at day 116 (Figure 9E–G). At day 120, the venous anastomosis was revised, and the flap was salvaged. The further course was uneventful, and bone healing was completed after 4 months.

### 2.2. All-Internal Segmental Bone Transport with Motorized Nails

External fixation has been a great addition to the treatment of musculoskeletal bone defects, but external fixators are related to a variety of problems and complications, including pain, pin tract infection, joint stiffness, interference with gait, discomfort and a host of aesthetic and psychological problems (Video S1).

**Figure 10:**
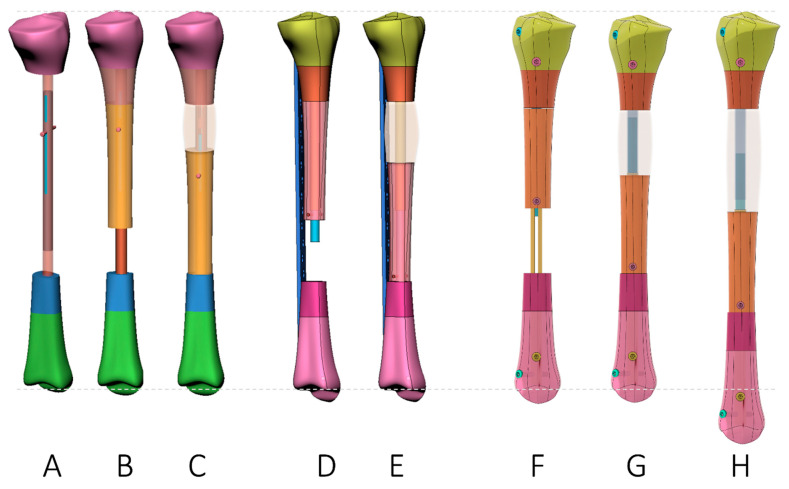
Different options for segmental bone transport with motorized nails. (**A**–**C**) Bone segment transport nail. (**D**,**E**) Plate-assisted bone segment transport (PABST). (**F**–**H**) MagicTube.

Motorized nails have been described for limb lengthening [57,58,59] (Video S2). The motor of the nail can be activated with mechanical energy by gait or, in more recent developments, with electrical energy either from a battery or magnetically induced with an actuating device. These devices have been adapted to bone defect situations where the lengthening nail functions as the driving motor for the bone segment for an ‘all-internal’ segmental bone transport. This can be reached with three different configurations (Figure 10, Table 1).

### 2.3. Bone Segment Transport Nails (STN)

These nails consist of a proximal and distal lock and a slotted bridging element. A central motorized piston is connected to the transport segment through the slot. With the movement of the piston, the connecting locking bolts glide in the slots of the central nail part and drive the bone segment [60,61] (Video S3).

### 2.4. Plate-Assisted Bone Segment Transport (PABST)

Here, a locking plate bridges the bone defect, and a conventional motorized lengthening nail transports the osteotomized bone segment into the defect [62,63] (Video S4).

### 2.5. MagicTube

The magic tube consists of a simple slotted tube that is slid over a motorized nail. The far end of the tube contains screw holes. This concept does not require an additional plate construct to bridge the proximal and distal main segments nor does it need a special segment transport nail since it is applicable to any lengthening nail (Videos S5 and S6). It also allows for additional lengthening if needed [64,65] (Videos S5 and S7).

## 3. Discussion

There are several techniques to choose from in the treatment of long-bone defects, as demonstrated above. The most significant difference is whether anatomical repair or reconstruction for continuity of the bone should be performed. The physical demand of the upper or lower limb must be taken into consideration, similar to the physical demand of the patient. Since the biomechanical characteristics of reconstruction techniques, such as fibula grafts of Masquelet, are generally inferior compared to anatomical repair, these techniques are more often used in the upper limbs. Because of the soft-tissue situation and the nearby anatomical structures in the upper limbs, which are potentially more at risk with distraction techniques with reduced time wearing an external fixator, this recommendation is supported from a safety and comfort perspective.

For lower-limb defects, restoration of the anatomical situation and biomechanical weight-bearing capacity seem essential, especially in younger patients. The Masquelet technique allows for quick reconstruction of bone continuity by missing cortex marrow differentiation. Fibula graft techniques have to double-fold the graft to gain a sufficient diameter if reconstruction, e.g., in the femur, is performed. This limits the length of the possible defects that can be reconstructed. Donor-site morbidity is also a relevant aspect to take into consideration when choosing the proper technique. Therefore, distraction osteogenesis in a classic, hybrid or all-internal manner shows advantages in the lower limbs. Classic and hybrid distraction devices are available around the globe and allow for the treatment of a great variety of defect morphologies and localizations in a cost-effective manner.

Nevertheless, external fixation is related to relative discomfort for the patient and a variety of possible complications, e.g., pin tract infections. To rule out these disadvantages, all-internal devices were developed that differ in the manner of activating the distraction device. Recently developed magnetically activated nails [66,67,68] have advantages over gait-activated systems [57], which are difficult to control (speed, stop) and cannot reverse direction. Magnetically or electrically activated motorized nails overcome these disadvantages. They allow for reliable control of the start and stop as well as the direction and speed.

All motorized segment transport nail systems benefit from internal components. All three technologies allow for the control of transport speed and direction. However, motorized nail systems have the disadvantage that they require a certain minimum length of the nail, which can be problematic in short proximal or distal fragments. In these circumstances, direct locking of the transport fragment may not be possible, and pulley systems (wire, plate) may need to be considered. Another disadvantage of magnetically motorized nails is the fact that the induction of energy is related to the distance between the external actuator and the internal receiver in the nail. In obese patients and limbs with a large soft-tissue envelope, the amount of energy transferred is less compared to normal-weight patients and slim limbs. Most of these patients with segmental defects also have some shortening. Additionally, there is frequently some shortening associated with debridement at the docking site. The MagicTube is the only device that allows for additional lengthening without further surgery just by continuing the segment transport. When STM was compared with PABST, it was shown that there are difficulties in maintaining alignment in short metaphyseal segments and fewer options to manage this when compared with the PABST procedure [61].

Due to the high costs of these devices, the availability and utilization of all-internal distraction devices are limited in health care systems worldwide.

## 4. Conclusions

The treatment of long-bone defects is challenging for the treating surgeon and the patient and his or her environment. In every case, the underlying pathology must be managed, such as infection eradication or resection of the tumor, before restoration of the defect can begin.

The physical demand of the patient and the localization of the defect influence whether repair or reconstruction techniques are used. Reconstruction techniques, such as Masquelet and vascularized fibula grafts, show good results and reduce the time of external fixation. Biomechanical disadvantages focus these techniques on the upper limbs. Repair techniques are mainly based on the work by Ilizarov [13,14,15,16], which can be performed in a classic all-external manner. Hybrid techniques reduce the time of the external fixator and show benefits at the time-point of docking. All-internal devices limit the complications of the external fixators but are limited in use because of the high cost.

Since the long process of restoration of a bone defect is demanding for the patient and the surgeon, the faith of the patient in the treating surgeon and the trustful cooperation of the patient and surgeon are as important for treatment success as the technique used.

## Figures and Tables

**Table 1:** Different options for all-internal distraction.

	Plate-Assisted Bone Segment Transport (PABST) [60,61]	Segment Transport Nails(STN) [62,63,64,65]	Nail-in-Nail System(MagicTube) [66,67]
Concept	Defect is bridged with a plate. Conventional motorized lengthening nail transports the osteotomized bone	These nails consist of a proximal and distal locked and centrally slotted bridging element. central motorized piston is connected to the transport segment	Bridging the proximal to the distal main segment is performed with a simple slotted tube with several locking holes
Advantage	Flexible use of commercially available standard implants (locking plate, lengthening nail). Allows for short proximal and distal main segments.	Consists of the one nail component only. No need for an additional plate.	Does not require an additional plate to bridge proximal and distal main segment No special segment transport nail needed Applicable to any lengthening nail Fragment size opposite the motor side can be as short as 3 cm Optional additional lengthening
Disadvantage	Difficulty achieving fixation in short metaphyseal segments Locking plate might interfere with soft tissues and/or nail Position of the piston locking holes might not be at the level of the transport segment, requiring a pulley system. Flexible pulley systems only allow pull, not push mode. Does not allow for sequential lengthening. Maximum transport length only limited by nail stroke	Difficulty maintaining alignment in short metaphyseal segments Less forgiveness in execution and very little chance of correcting as the transport is underway Does not allow for sequential lengthening Additional surgical procedure required when transport length exceeds 70 mm	Difficulty maintaining alignment in short metaphyseal segments Adds to the thickness of the lengthening nail Not FDA approved or CE marked Maximum transport length only limited by nail stroke

## Supplementary Materials

The following supporting information can be downloaded at: https://www.mdpi.com/2077-0383/12/19/6283/s1, Video S1: EF. Video S2: motorized nails for limb lengthenig, Video S3: Bone segment transport, Video S4: plate assisted bone segment transport, Video S5: Magic tube, Video S6: Magic tube.
